# Spliceosome-Associated microRNAs Signify Breast Cancer Cells and Portray Potential Novel Nuclear Targets

**DOI:** 10.3390/ijms21218132

**Published:** 2020-10-30

**Authors:** Shelly Mahlab-Aviv, Keren Zohar, Yael Cohen, Ayelet R. Peretz, Tsiona Eliyahu, Michal Linial, Ruth Sperling

**Affiliations:** 1 Department of Biological Chemistry, Institute of Life Sciences, The Hebrew University of Jerusalem, Jerusalem 91904, Israel; shelly.mh@gmail.com (S.M.-A.); keren.zohar@mail.huji.ac.il (K.Z.); tsiona.e@mail.huji.ac.il (T.E.); 2 Department of Genetics, Institute of Life Sciences, The Hebrew University of Jerusalem, Jerusalem 91904, Israel; Yael.cohen24@mail.huji.ac.il (Y.C.); ayelet.peretz@mail.huji.ac.il (A.R.P.)

**Keywords:** RNA-seq, antimiR, oncomiR, carcinogenesis, ncRNA, metastasis, nuclear miRNAs

## Abstract

MicroRNAs (miRNAs) act as negative regulators of gene expression in the cytoplasm. Previous studies have identified the presence of miRNAs in the nucleus. Here we study human breast cancer-derived cell-lines (MCF-7 and MDA-MB-231) and a non-tumorigenic cell-line (MCF-10A) and compare their miRNA sequences at the spliceosome fraction (SF). We report that the levels of miRNAs found in the spliceosome, their identity, and pre-miRNA segmental composition are cell-line specific. One such miRNA is miR-7704 whose genomic position overlaps HAGLR, a cancer-related lncRNA. We detected an inverse expression of miR-7704 and HAGLR in the tested cell lines. Specifically, inhibition of miR-7704 caused an increase in HAGLR expression. Furthermore, elevated levels of miR-7704 slightly altered the cell-cycle in MDA-MB-231. Altogether, we show that SF-miR-7704 acts as a tumor-suppressor gene with HAGLR being its nuclear target. The relative levels of miRNAs found in the spliceosome fractions (e.g., miR-100, miR-30a, and let-7 family) in non-tumorigenic relative to cancer-derived cell-lines was monitored. We found that the expression trend of the abundant miRNAs in SF was different from that reported in the literature and from the observation of large cohorts of breast cancer patients, suggesting that many SF-miRNAs act on targets that are different from the cytoplasmic ones. Altogether, we report on the potential of SF-miRNAs as an unexplored route for cancerous cell state.

## 1. Introduction

MicroRNA (miRNAs) are small, ~22 nt long, non-coding RNA molecules implicated in defining cell identity and regulating signaling pathways. Variations in miRNA expression have been linked with numerous human diseases including cancer [1,2]. The main studied role of miRNAs in mammals is in inhibiting translation and negatively controlling gene expression. This is mediated by base-pairing of miRNAs primarily to the 3’-UTR of the target mRNA transcripts in the cytoplasm [3,4,5,6,7]. In humans, many miRNA genes are located in gene introns. The canonical biogenesis of intronic miRNAs from Pol II transcripts involves two main steps: The first step occurs in the nucleus by the microprocessor, whose key proteins are DGCR8 and Drosha. DGCR8 binds to the RNA molecule, while Drosha, an RNase III type enzyme, cleaves the primary (pri) miRNA transcript into a precursor (pre) miRNA stem-loop molecule of 70–80 bases [8,9,10,11,12]. The second step occurs after the export of the pre-miRNA to the cytoplasm [13] when it is cleaved by Dicer, yielding a mature miRNA, which is loaded on the RNA Induced Silencing Complex (RISC) [3,4,14]. The RISC-bound miRNAs act in targeting mRNAs in the cytoplasm [7]. According to miRBase [15] over 1900 miRNA genes are found in the human genome, yielding ~2600 mature miRNAs. It was estimated that miRNAs control ~60% of all human genes [3]. Alternation in the quantity of miRNAs expression has the potential to perturb cellular pathways leading to human diseases such as neurodegenerative diseases, chronic inflammation, metabolic disorders, and cancer onset and progression [1,2,15,16].

The finding of mature miRNAs in the nucleus [17,18,19,20,21], suggested nuclear functions for miRNA in addition to the classical cytoplasmic ones [22,23]. The active process of shuttling of miRNAs from the cytoplasm to the nucleus were demonstrated [24,25]. The function of miRNAs in the nucleus are not yet fully understood [21]. However, the involvement of miRNA in a number of processes, such as the regulation of non-coding RNAs [26,27,28,29,30], transcriptional silencing [31,32], activation [33,34,35,36], and inhibition [32] were reported. Furthermore, analysis of miRNA-mRNA-AGO interactions, revealed substantial AGO-miRNA mapping to intronic sequences [37].

A large fraction of miRNA genes are located in the introns of coding genes, while many are expressed from their own Pol II promoters [6,38,39]. For most intronic miRNAs, the mRNA and such miRNA can be expressed from the same primary transcript. However, when the pri-miRNA is located in an exon or overlaps a splice site, at any specific time only the mRNA or the miRNA can be generated from the single transcript. In the case of alternative splicing (AS), the expression of both the mRNA and the miRNA from the same transcript is possible [39]. Furthermore, several studies showed links between splicing and miRNA processing [25,40,41,42,43,44].

Splicing and AS play a major role in the regulation of gene expression in mammals [45]. Furthermore, changes in AS occur in many human diseases including cancer [46,47,48]. Splicing occurs in the cell nucleus within a huge (21 MDa) and highly dynamic machine known as the supraspliceosome [49,50]. It is composed of four native spliceosomes, which are connected by the pre-mRNA [51,52]. The entire repertoire of nuclear pre-mRNAs, independent of their length and number of introns, is individually assembled in supraspliceosomes [49,50]. The supraspliceosome offers coordination and regulation of pre-mRNA processing events. Thus, it is involved in all nuclear processing activities of pre-mRNAs [49,50]. Furthermore, miRNAs were found within the endogenous spliceosome [44,53,54,55], where a cross-talk between pre-mRNA splicing and miRNA processing was demonstrated [44,55].

A recent study on the composition of small ncRNA (<200 nt) within the spliceosomal fraction (SF) from HeLa cells identified about 200 miRNA sequences [54], several that are exclusively expressed in the SF. One of these SF-miRNAs that is exclusively expressed in SF is miR-7704. miR-7704 was shown to be a negative regulator of HAGLR (HOXD Antisense Growth-Associated Long Non-Coding RNA) expression in HeLa cells [54]. While about two-thirds of these SF-miRNA sequences were identified as mature miRNAs, the rest represent segments derived from pre-miRNAs. Many of these sequences are not harbored in introns. These findings indicate that the presence of miRNA sequences in the endogenous spliceosome is not only due to biogenesis, and it further suggests novel functions for miRNAs within the endogenous spliceosome.

In this study, we focused on SF-miRNAs in the human cell-lines of breast origin. We analyze the SF-miRNA composition and unique properties for two breast cancer-derived cell-lines (MCF-7 and MDA-MB-231) and a non-tumorigenic cell-line (MCF-10A). These cells are often used as cellular models for breast cancer. We investigated miR-7704 and show that it negatively regulates the expression of lncRNA HAGLR, which plays a role in the development and progression of multiple cancers [56]. The potential role of SF-miRNAs in defining the cellular state of cancer is discussed.

## 2. Results

### 2.1. Isolation of Spliceosomal RNA from Breast Cell-Lines

**Figure 1:**
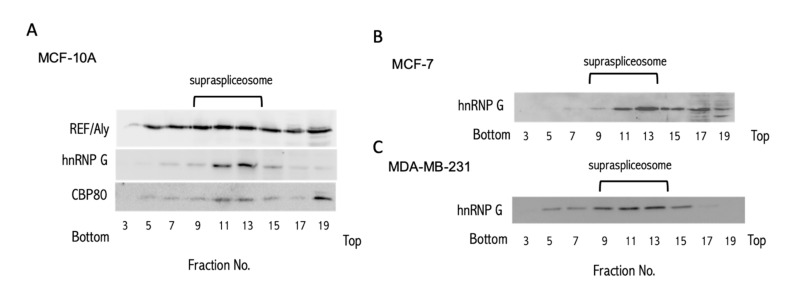
Splicing factors mark the supraspliceosome fraction (SF) in breast cancer cells. WB analysis of the distribution of splicing factors across the glycerol gradients. Nuclear supernatants enriched for SF were prepared from MCF-10A (**A**), MCF-7 (**B**), and MDA-MB-231 cells (**C**), and were fractionated in 10–45% glycerol gradients. Tobacco mosaic virus (TMV) was used as a size marker for the sedimentation. Aliquots from odd gradient fractions were analyzed by WB using anti-hnPNP G, anti-REF/Aly and anti CBP80 antibodies. Supraspliceosomes peak in fractions 9–13 (200S). We used the distribution of hnRNP G (42 kDa) to follow the enrichment of the supraspliceosome fractions across the gradient.

The identification of hundreds of miRNA sequences in SF from HeLa cells prompted us to search for spliceosomal miRNAs in cells from a different origin. To this end, we have chosen the breast cell-line MCF-7, an estrogen-dependent ductal carcinoma, and the MDA-MB-231, which is a highly invasive and metastatic estrogen-independent adenocarcinoma. We also tested MCF-10A, a mammary epithelial cell-line, as a model for healthy cell function. We prepared nuclear supernatants enriched with supraspliceosomes under native salt conditions, and fractionated each on glycerol gradients as previously described [51] (see Materials and Methods). Notably, the isolation protocol preserves the higher-order splicing complexes as shown by electron microscopy [51,57]. The supraspliceosomes sediment at 200S with the splicing factors, hnRNP G, and REF/Aly, as well as the cap-binding protein CBP80. We used these components to locate the position of the supraspliceosome in these gradients [58,59,60]. Figure 1 presents the results of Western blot (WB) analyses across the glycerol gradient performed on samples from each of the breast cell-lines, using antibodies directed against genuine supraspliceosomal components.

Next, we extracted small RNA (<200 nt) from the spliceosomal fraction (SF, fractions 9–12, Figure 1) of each of the breast cell-lines and used the RNA in the SF to construct a barcoded library of small RNA for further sequencing, as previously described [54]. We created three different libraries for biological triplicates from each cell-line. The statistical summary of the RNA-seq libraries is shown in Supplementary Table S1. Alignment of these SF sequences to the human transcriptome revealed a complex collection of RNA species, including pre-miRNAs, small nucleolar RNAs (SNORDs) [61], intronic sequences, and more. In this study, we only consider reads that are aligned to the hairpin precursor of the primary miRNA as determined by miRBase [15]. These sequences are referred to as SF-miRNAs. We refer to the levels of miRNA found in the spliceosome fraction as an SF-miRNA expression level.

### 2.2. Changes in Expression Levels of SF-miRNA Sequences in Breast Cancer Cells

Sequencing and alignment to all transcriptome and miRNA collections revealed a large complexity of the miRNA sequences, ranging over 2–3 orders of magnitude (Supplementary Table S2).

**Figure 2:**
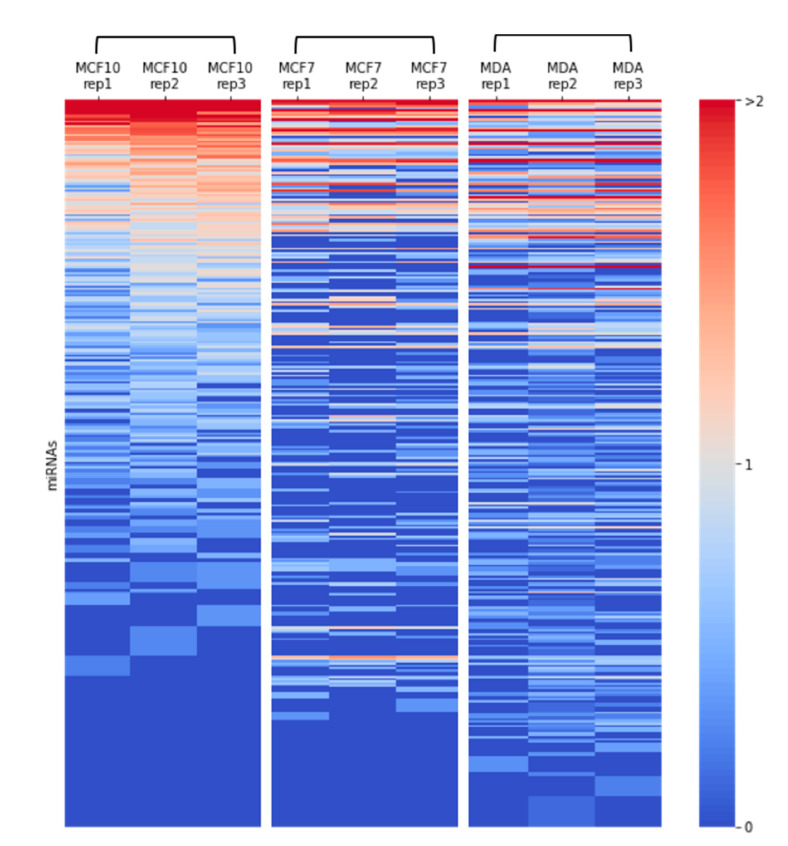
Heatmap of miRNA expression in breast cancer cells. Expression of SF-miRNAs in triplicates from each of the breast cancer cell-lines: MCF-10A, MCF-7, and MDA-MB-231, are presented. Color code represents the amount of reads in a logarithmic scale (log10). All miRNAs that are analyzed have ≥10 reads in any specific cell type, a minimal read length of ≥17. The data source is in Supplemental Table S2.

The sequences were aligned to 397, 195, and 246 different entities for MCF-10A, MCF-7, and MDA-MB-231 cells, respectively (≥2 reads per cell-line). However, for increasing the reliability, we restricted the analysis to a subset of higher confidence sequences with a mandatory read length of ≥17, and at least 10 reads that are found in SF from each of the cell-lines. Figure 2 shows a heatmap of the quantities of the different SF-miRNAs from each of the RNA-seq libraries (3 cell-lines in triplicates). The correlation of the miRNAs identified for each of the 9 miRNA profiles is shown in Supplemental Figure S1. The triplicates from MCF-10A show the strongest correlation, and the correlations of the miRNA profile for MCF-7, and MDA-MB-231 are less distinctive, suggesting a resemblance in the miRNA expression profiles for these two cancerous cell-lines.

**Figure 3:**
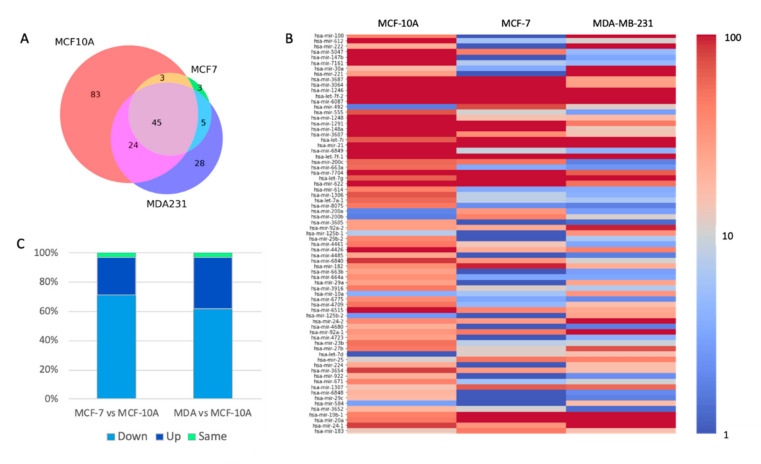
Changes in expression of SF-miRNAs among breast-origin cell-lines. (**A**) Venn diagram showing the partition of different SF-miRNAs among three breast cancer cell-lines. Source data from Supplementary Table S2. (**B**) Following normalization of reads and testing statistics for differential expression across the three cell-lines, a significant list of 73 SF-miRNAs is shown, colored by the amounts of normalized reads (log 10 scale). All listed SF-miRNAs met the statistical adjusted *p*-value of <0.05 and are ranked by their statistical significance. (**C**) Partition of the 73 SF-miRNA according to the 3 possible expression trends composed of up (U), down (D), and S (same). Paired expression trend is the trend for MCF-7 versus MCF-10A and for MDA-MB-231 versus MCF-10A. The dominant observed trend is marked as D, followed by U. MDA-MB-231 marked as MDA, or MDA231. The data source is in Supplemental Table S3.

Based on the thresholds used for reliable reads, we identified 155, 56, and 102 SF-miRNAs in MCF-10A, MCF-7, and MDA-MB-231 cells, respectively. The unified list includes 191 SF-miRNAs (Supplementary Table S2). Figure 3A presents a Venn diagram of the overlap of SF-miRNAs observed among the tested cell-lines. We find that 45 miRNA sequences are expressed in all three cell-lines. The highest expressing miRNAs include miR-6087, miR-21, miR-20a, and let-7g (Supplemental Table S2). When testing the miRNAs that are shared in both MCF-10A and MCF-7, an additional 3 miRNAs were found: miR-3652, miR-5047, and miR-200c. Additional 24 SF-miRNAs are shared between MCF-10A and MDA-MB-231 (e.g., miR-100, miR-222, miR-221, and miR-30a). For the two cancerous cell-lines (MCF-7 and MDA-MB-231) additional 5 SF-miRNAs were found (e.g., miR-492, miR-200b). Figure 3A further illustrates that many of the miRNAs are exclusively expressed in a specific cell-line. A total of 83, 3, and 28 are expressed solely in MCF-10A, MCF-7, and MDA-MB-231, respectively.

For testing the trend in expression of each SF-miRNA, we analyzed the differential expression profile by the statistical significance of the expression results following normalization (DEseq2, Supplemental Table S3, see Materials and Methods). Figure 3B shows the results by the level of expression (in log scale) represented as a colored heatmap. The miRNAs are sorted according to the differential expression of significant findings. Specifically, the most significant differential expressed SF-miRNA across all three cell-lines is miR-100 (adjusted *p*-value = 4.65E-65). Only miRNAs with adjusted *p*-value < 0.05 are listed, resulting in 73 statistically significant differentially expressed miRNAs (Supplemental Table S3).

Figure 3C presents a partition of expression trends of these 73 listed SF-miRNAs (Figure 3B). To test whether the cell-lines are signified by a clear trend in expression, we compared MCF-7 and MDA-MB-231 to the reference of MCF-10A. For simplicity, we converted the expression levels to discrete trends marked as Up (U), Down (D), and Same (S). A trend marked as the Same indicates a stable expression (±20%). The dominant trend among all 73 SF-miRNAs shows a decrease (D) in expression when comparing each of the cancerous cell-lines MCF-7 and MDA-MB-231 to MCF-10A cells (52 (71%) and 45 (62%) SF-miRNAs, respectively). Another group of the SF-miRNAs shows an increase (U) in expression when comparing each of the cancerous cells MCF-7 and MDA-MB-231 with MCF-10A (19 (26%), and 26 (36%) SF-miRNAs, respectively) (Figure 3C). Only a negligible fraction of the listed SF-miRNAs shows a stable expression (3%).

Table 1 presents the top 25 SF-miRNAs ranked by their expression levels in the three cell-lines, including the genomic position of the identified SF-miRNAs (see also Supplementary Table S3). For some miRNAs (miR-1291, miR-1248, miR-3607), the genomic location overlaps with SNORA/D sequences. For 8 of the listed miRNAs, the sequences are located in introns of coding genes. Numerous miRNA-aligned sequences are intergenic, while the majority of the other miRNAs are associated with lncRNAs, including transcripts that account for clusters of neighboring miRNAs (Table 1). Several SF-miRNAs (e.g., miR-5047 and miR-3064, Table 1) are miRNAs that belong to the same genomic cluster. We conclude that among the statistically significant differentially expressed SF-miRNAs, several are in the vicinity of ncRNA and in regions with local genomic miRNA clusters.

### 2.3. Changes in the Segmental Regions of SF-miRNAs in Breast Cancer Cell Lines

**Figure 4:**
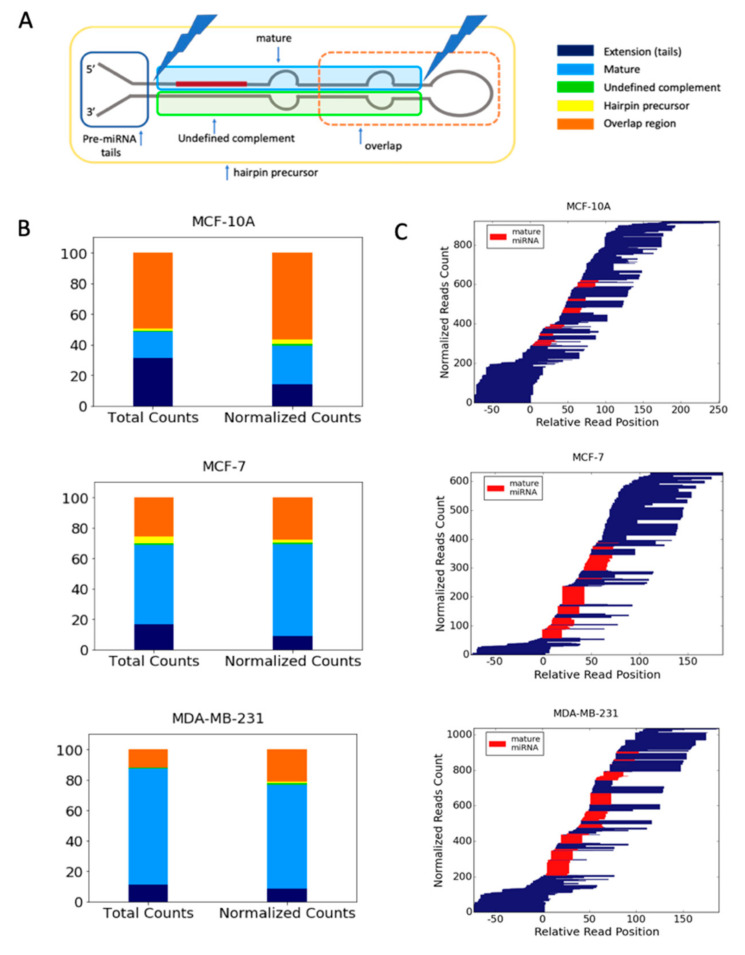
Partition of the SF-miRNAs to segmental pre-miRNA regions. (**A**) A schematic view of an miRNA prototype, where the different regions of the HP-miRNA are listed according to their positions with respect to the pre-miRNA major processing sites. An example for an overlap region, defined as reads that cross known segmental boarders, is indicated. (**B**) The relative counts of reads mapped to each of the predefined regions for each of the three cell lines. The relative counts of reads to each of the pre-miRNA regions are color-coded as in A. Total counts of reads (left) and normalized counts (right, the relative abundance of reads aligned to a specific miRNA sums to 1) by each miRNA are shown for MCF-10A, MCF-7, and MDA-MB-231. (**C**) Blocks of reads from the same starting points reflect non-randomized cleavage sites. The 5p and 3p mature miRNAs are colored red, and the width of the bars is proportional to the number of reads. The position and alignment of the mature miRNAs is according to the miRbase annotations. All miRNAs that are analyzed have an average minimal number of reads per cell-line (≥3), and a minimal read length of ≥17. Data source is in Supplemental Table S4.

To further characterize the properties of the SF-miRNAs we assessed the segmental composition of each identified miRNA. We observed that the SF-miRNA sequences are not limited to mature miRNAs. We classified the SF-miRNA sequences according to the segmental regions to which they align (Figure 4A). These regions are based on the complete hairpin precursor miRNA (HP-miRNA) sequence (as determined by miRBase), mature miRNA (either derived from the 5p or the 3p); undefined complement (i.e., a complementary sequence of mature miRNA where there is no experimental evidence in miRBase), and miRNA extension (tails of <50 nt of genomic extended sequences from the mature miRNA/complementary sequence). Reads that do not reside within these predefined regions, but overlap any two or more regions, are categorized as an overlapping region. It is likely that all the segments from the pre-miRNA may act by base-pair complementarity.

Figure 4B shows the distribution of reads among these disjoined categories in the tested cell-lines (see also Supplemental Table S4). It can be seen that each cell-line provides a different partition of the miRNA segmental regions. For MCF-10A cells, the mature miRNAs account for only ~25% (length size ~22 nt), while it dominates the aligned sequences in MDA-MB-231 cells. In the MCF-10A cell-line, ~14% of the aligned reads represent extension sequences while the majority (~57% of normalized counts) belong to overlapping regions. However, in the MCF-7 and MDA-MB-231 cells, the percentage of mature miRNA is ~60% and 68%, respectively (Figure 4B and Supplemental Table S4). The percentage of overlap regions is the lowest for MDA-MB-231 (21%). The remaining miRNA aligned sequences belong to miRNA extension categories that are 14% and 9% in MCF-10A and MDA-MB-231, respectively (Figure 4B). Notably, the distribution along the HP-miRNA sequence is quite wide as demonstrated in Figure 4C. The scheme shows that the amounts, length, and segmental profiles of the pre-miRNA genomic sequences vary substantially among the three cell-lines. The mature miRNAs dominate most of the SF-miRNAs from MDA-MB-231 cells. However, some miRNAs display a complex segmental composition. For example, the aligned reads for miR-151a, miR-181a-1, miR-146a, and miR-98 were split between mature and overlap regions. Segmental regions of SF-miRNAs are listed in Supplemental Table S4. The changes in the partition of the miRNA segmental regions between the breast cell-lines, and especially between the breast cancerous cells and the non-malignant breast cell, portray the potential of SF-miRNAs to recognize and attenuate their nuclear targets in a cell-specific manner.

### 2.4. Negative Correlation Between the Expression of SF-miR-7704 and the Oncogenic lncRNA HAGLR

**Figure 5:**
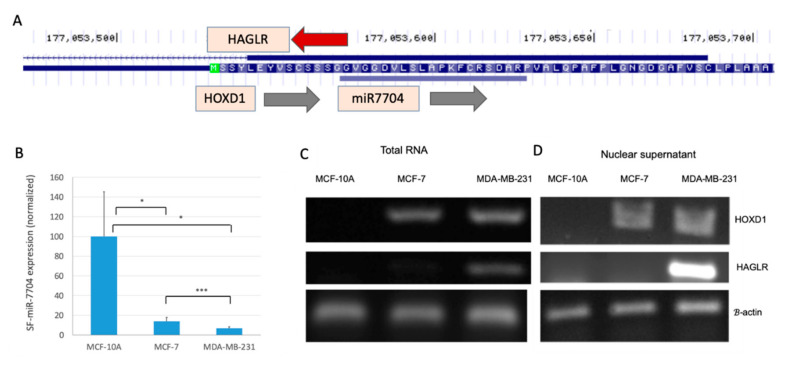
Inverse correlation between the expression of miR-7704 and HAGLR. (**A**) UCSC Genome browser view for miR-7704 indicates the overlap with HOXD1 and the lncRNA HAGLR. (**B**) Average expression levels and standard error of the mean (SEM) of miR-7704 from the SF from MCF-10A, MCF-7 and MDA-MB-231 cells. Pair statistics are marked for <0.1 (*) and <0.001 (***). Comparison of the results of RT-PCR assays of HOXD1 and HAGLR expression as measured from total RNA (**C**) and nuclear RNA (**D**) in the three tested breast cell-lines. Results shown are representative RT-PCR. The identity of the extracted bands was confirmed by DNA sequencing.

Inspecting the SF-miRNAs in HeLa cells showed that the collection of SF-miRNAs is different from that of the cytosolic miRNAs [54]. Furthermore, many of the SF-miRNAs were derived from intergenic regions (e.g., Table 1). Thus, it is most likely that the mode of action of the miRNA sequences in the spliceosome is different from that of the classical translation suppression that specifies cytosolic miRNAs. To test this possibility, we focused on SF-miR-7704 that was previously shown to negatively regulate the lncRNA HAGLR [54]. Here we analyzed the changes in expression of miR-7704 and HAGLR in the three breast-originated cell-lines. The genomic position of miR-7704 with respect to the neighboring genes is illustrated in Figure 5A.

We show that the level of expression of SF-miR-7704 varies in each of the tested cell-lines with maximal expression in the MCF-10A and a reduced level of expression in the MDA-MB-231 cells (Figure 5B). RT-PCR analyses of the expression of HAGLR and HOXD1 (Figure 5C,D) confirm the relative changes in expression in the breast-originated cell-lines. Figure 5C,D demonstrate that the expression level of HAGLR is hardly noticeable in MCF-10A cells, but its expression is evident in the MDA-MB-231 cells. The high expression level of HAGLR is negatively correlated with the expression level of miR-7704. These findings were repeated when RNA was extracted from the cells or from isolated nuclei. The expression level of HOXD1 (which is on the same strand as miR-7704) is lowest in MCF-10A and will not be further discussed. It should be noted that HAGLR was proposed to be involved (directly or indirectly through miRNA binding) in apoptosis, cell invasion and proliferation characteristics in many cancer types [56].

### 2.5. Manipulating the Expression Level of miR-7704 Dictates HAGLR Expression Level

To test the direct link between the expression of miR-7704 and HAGLR, we transfected (in triplicates) each of the breast cell-lines with an anti-miR-7704 inhibitor. We next extracted nuclear RNA from each of the transfected cell-lines and measured the expression levels of miR-7704 and HAGLR by quantitative-PCR. Cells transfected with non-silencing anti-miRNA and untreated cells were used as controls (see Materials and Methods).

**Figure 6:**
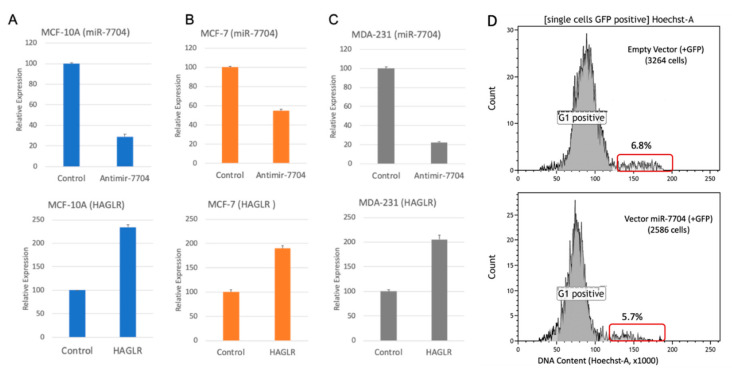
Manipulating SF-miR-7704 levels. (**A**–**C**) Inhibition of miR-7704 expression upregulates the expression of HAGLR. Results of quantitative real time PCR analysis of the effect of the inhibition of miR-7704 on the nuclear expression of HAGLR in MCF-10A (**A**), MCF-7 (**B**), and MDA-MB-231 cells (**C**) are shown. Transfection with Anti-miR-7704 inhibitor resulted in the down-regulation of miR-7704 (upper panel), and an increase in the expression level of HAGLR mRNA (lower panel) relative to the control. For the control we used the non-silencing Anti-miR (see Materials and Methods). The average of 3 independent biological preparations are shown, including the standard error. The expression levels of HAGLR was normalized to the internal control of ß-actin expression from the same preparation. (**D**) The FACS analyses are based on staining cells with Hoechst to quantify the DNA content following transfecting the MDA-MB-231 cells with miR-7704 or with its empty vector. The sorting is based on co-expression of GFP (488 nm; positive cells). The results from the overexpression with the empty vector (top) and the of same vector with miR-7704 (bottom) are shown. The fraction of the cells which accounts for an excess of DNA (>2 n; S/G2/M in cell cycle) are marked by a red frame.

As shown in Figure 6A, the transfection of MCF-10A with an anti-miR-7704 inhibitor reduced the level of nuclear miR-7704 to 30% as compared with the non-treated cells, while a non-silencing anti-miR had a negligible effect. The decrease in the level of nuclear miR-7704 is followed by >2 folds of upregulation in the level of lncRNA HAGLR (Figure 6B, lower panel). When the same protocol was applied for MCF-7 cells (Figure 6B), transfection with anti-miR-7704 reduced the level of nuclear miR-7704 in the cells to 55% of its level in non-treated cells. In these cells, the upregulation in the level of HAGLR was ~2 folds (Figure 6B, lower panel). Transfection of MDA-MB-231 cells with anti-miR-7704 reduced the level of miR-7704 to ~20% and its original level, resulting in a 2-fold increase in the level of HAGLR (Figure 6C). We conclude that miR-7704 negatively regulates the expression of the oncogenic lncRNA HAGLR, known to play a role in the development and progression of multiple cancers [56].

To test whether artificial elevation of miR-7704 has the potential to alter the cell properties, we analyzed the unsynchronized cells by quantifying whether there is a shift in the fraction occupied by the cell cycle stages (divided to G0/G1 and G2/S/M) as a proxy for alteration in cell division. We compared the changes in the G2/S/M phase relative to the basal level of miR-7704. MDA-MB-231 cells were transfected with a plasmid expressing has-miR-7704 (see Materials and Methods) and empty vector as a control. As miR-7704 overexpression is accompanied by GFP expression, we used the fluorescence signal to sort cells that were transfected (i.e., with elevated levels of miR-7704). Figure 6D shows that as a result of miR-7704 overexpression in MDA-MB-231 cells, the fraction of cells in the G2+S was reduced (from 6.8% to 5.7%, Figure 6D). A modest reduction (9–16%, 4 transfection experiments) in cells at the S/G2/M stages for the miR-7704 overexpression relative to the empty vector was measured. The increase in the level of miR-7704 led to an attenuation in dividing cells. We thus suggest that increased levels of miR-7704 altered the dynamic of the cell cycle in MDA-MB-231 cells. Recall that miR-7704 is among the SF-miRNAs which are uniquely found in the nucleus.

### 2.6. Comparing the Differential Expression of SF-miRNAs in Breast Cell-Lines

Based on the detailed analysis showing that the expression level of miR-7704 is cell-specific, and that HGLAR lncRNA is its nuclear target, we set to test the SF-miRNAs that are signified by differential expression among the cell lines.

**Figure 7:**
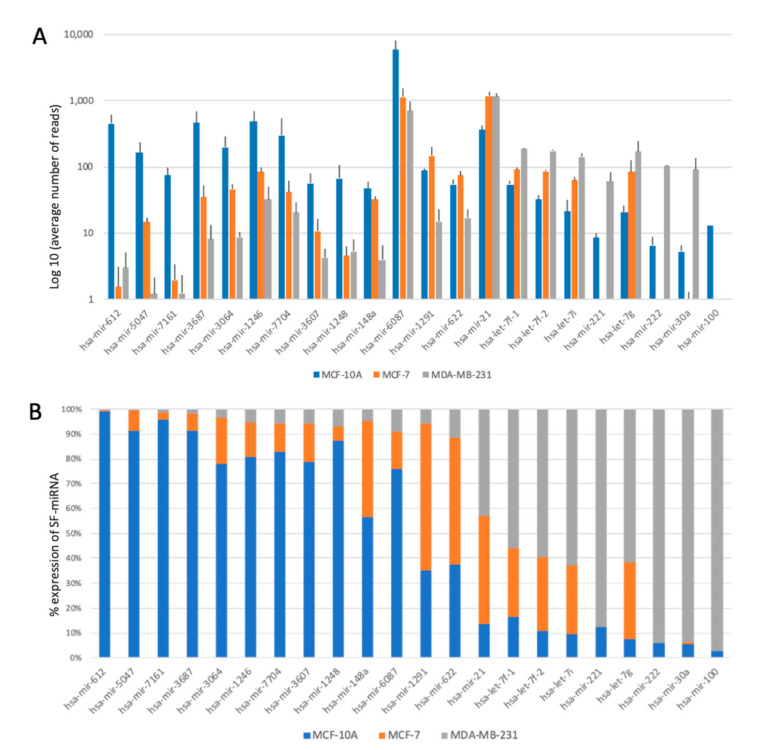
Changes in the expression of top SF-miRNAs with breast cancer tumorigenicity. (**A**) Changes in the expression level of the top 22 SF-miRNAs in the breast-originated cell-lines (log scale). (**B**) The partitions for each miRNA within the three cell-lines is shown. MCF-10A (blue), MCF-7 (orange), and MDA-MB-231 (grey). The data source is in Supplemental Table S3.

Figure 7A shows the log-scale expression of the top SF-miRNAs in the analyzed cell lines. Note that for a number of SF-miRNAs, orders of magnitude changes in expression level are recorded across the tested cells. Figure 7B shows the partition of expression for each miRNA among the three cell-lines. The majority of changes in expression of SF-miRNAs can be classified into two groups, those showing a low abundance in the cancerous cells (MCF-7 and MDA-MB-231) compared to the MCF-10A cell-line, and those showing the opposite trend, with the lower expression in the non-cancerous breast cell-line MCF-10A. Notably, the most significant expression differences are associated with MCF-10A and MDA-MB-231 cells. The expression levels of SF-miRNAs relative to the expression in the cytosol shows some discrepancy. For hsa-let-7 members, the level in the SF is much higher with respect to the negligible levels in the cytosol [62]. Furthermore, miR-221 and miR-222 were not detected at all in the spliceosome fraction of MCF-7, and their expression is restricted to spliceosome fractions of MDA-MB-231 cells (Figure 7). Monitoring the levels of miR-221 and miR-222 in the cytosols indicates that their level in MDA-MB-231 cells is only 2–3 fold higher relative to MCF-7 cells. Interestingly, an extreme ratio of miR-100 levels in the SF context was monitored for MDB-MB-231 relative to MCF-10A. However, an opposite profile was reported for cellular miR-100. The level of expression of miR-100 in the cytosol of MDB-MB-231 cells is low, and following its overexpression cell migration and invasion were inhibited [63].

**Figure 8:**
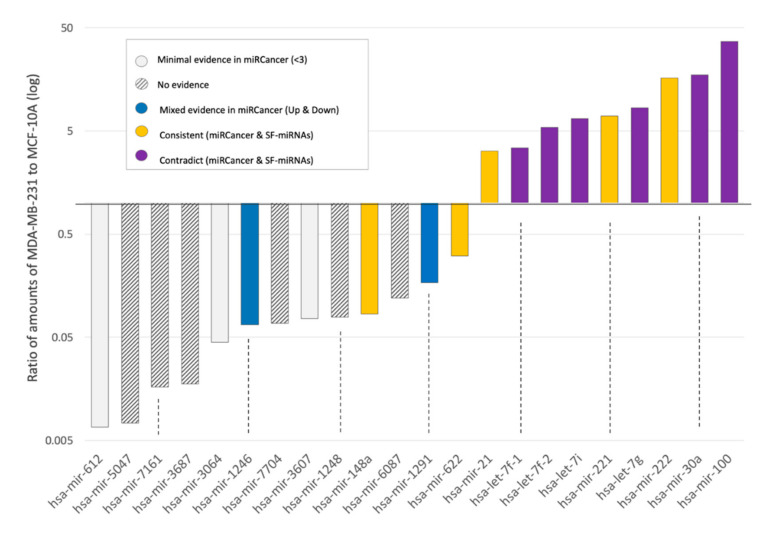
Expression level of SF-miRNAs in breast cancer cell-lines with respect to miRCancer annotations. Ratio of expression of SF-miRNAs in MDA-MB-231 to MCF-10A (log scale). A total of 22 differentially expressed SF-miRNAs are listed with a minimal expression levels of >50 reads (sum of MCF-10A and MDA-MB-231 expression reads). The color signifies the comparison according to the consistency with miRCancer (yellow—consistent trend; grey stripes—not available; blue, mixed trend; purple, opposite trend; white, minimal support (defined as miRNA with only 1–2 reported publications).

We compared the fold change and the trend in expression of SF-miRNAs between these two cell-lines (Figure 8). We report on 22 miRNAs that reach a joint expression level >50 normalized reads and were shown by their differential expression values to be significant (Supplementary Table S3). We assess the match with information derived from the current literature on breast cancer as annotated by miRCancer [64]. miRCancer compiles publications on cancer and annotates each miRNA by the relative expression in cancerous versus healthy samples (marked Up/Down). We show that for a number of the tested SF-miRNAs, the expression trend is consistent with that reported by miRCancer (e.g., miR-21, miR-148a, green). In contrast, the expression of miR-100, miR-30a, let-7g, let-7i, let-7f-1, and let-7f-2 is suppressed in breast cancer samples, while the expression level measured in the SF for these miRNAs is higher in the cancerous cell-line (MDA-MB-231) relative to MCF-10A. These findings argue that these SF-miRNAs are likely to act on nuclear targets, which are different from the known cytoplasmic ones. For miRCancer reports on the selected SF-miRNA, see Supplemental Table S5.

### 2.7. Inverse Expression Trend of SF-miRNAs and Total miRNAs from Breast Cancer Biopsies

The phenomenon of inverse directionality in expression of the SF-miRNAs in the breast cell-lines compared with the data collected from miRCancer is observed for 6 miRNAs (out of 22 listed SF-miRNAs). For another 9 SF-miRNAs, miRCancer provides no or poorly supported information (<3 publications, Figure 8). This shortage of data prompted us to investigate the relative expression of these miRNAs from breast cancer clinical samples. To this end, we applied a Kaplan–Meier (KM)-plotter for the breast cancer miRNAs collection [65].

**Figure 9:**
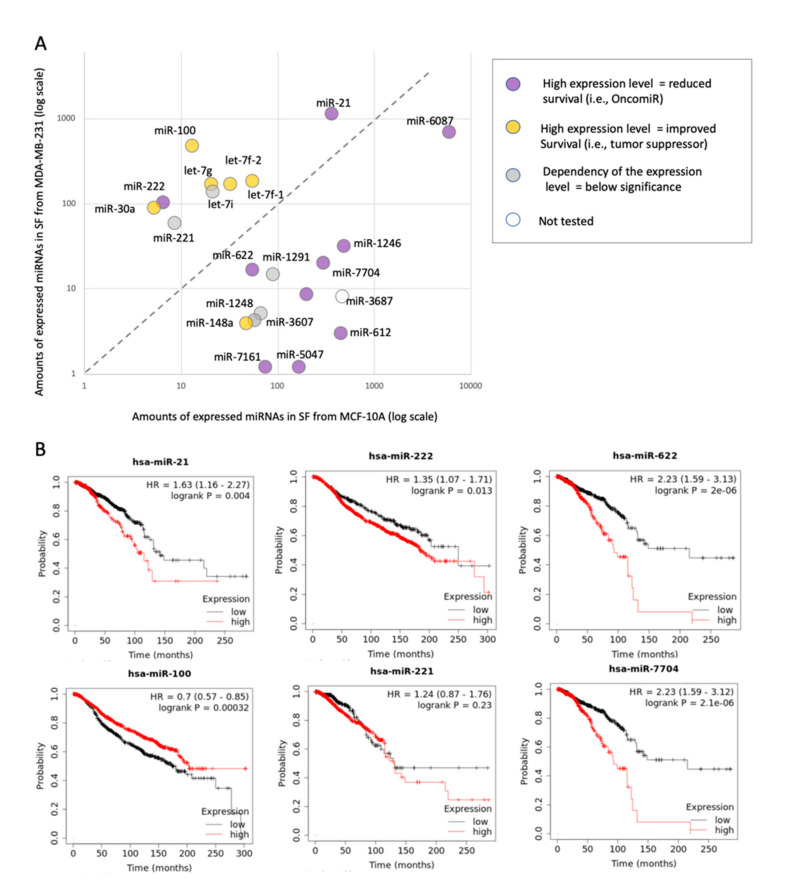
Expression of SF-miRNAs in different cell-lines vis-à-vis the patient’s overall survival rate. (**A**) Scatter plot of the SF-miRNAs expression in non-cancerous MCF-10A cells (x-axis) and metastatic MDA-MB-231 cells (y-axis) is presented. A total of 22 differentially expressed SF-miRNAs are listed with a minimal expression levels of >50 reads (sum of MCF-10A and MDA-MB-231 expression reads). The results of the Kaplan–Meier (KM) survival plots are color coded with miRNAs that are oncogenic (purple), protective (yellow), and statistically insignificant (grey). Each tested miRNA is colored by the survival rate from breast cancer patients extracted from the TCGA (1062 samples) and METABRIC (1262 samples) projects. The KM survival plots analyze the survival (in months) associated with high and low expression quartiles of the selected miRNAs. Analysis is completed for 21 of the 22 SF-miRNAs (see Supplemental Table S6). (**B**) Representative SF-miRNAs analyzed by KM survival plots with respect to the calculated hazard ratio (HR, 95% confidence intervals) and logrank with a *p*-value < 0.05.

Figure 9A shows the relative expression of all 22 SF-miRNAs (as in Figure 8) in the non-malignant MCF-10A cells and the metastatic MDA-MB-231 cells. The SF-miRNAs above the diagonal (Figure 9A) are specified by a higher expression in the metastatic cells (MDA-MB-231) versus the non-malignant healthy-like cells (MCF-10A). The color of each SF-miRNA indicates the impact of the miRNA expression on the patient survival as analyzed from two large breast cancer cohorts: the TCGA (1062 samples) [66] and METABRIC (1262 samples) [67]. The expression of each miRNA in each of the cohorts was normalized and split into high and low quantiles. Figure 9B shows representative miRNAs by their KM survival plots. For each miRNA, the hazard ratio (HR, 95% confidence intervals) and the logrank *p*-value were calculated. Information was available for 21 of 22 SF-miRNAs (Supplemental Table S6). The expression level of SF-miR-21 is elevated in MDA-MB-231 cells compared to the non-malignant MCF-10A, and it agrees with its malignancy index as tumorigenic (purple), while the expression level of SF-miR-148a is decreased with malignancy, in agreement with its malignancy index annotation as a tumor suppressor (yellow).

However, we found that for 5 SF-miRNAs whose expression level increased from the healthy to metastatic cell models (miR-100, let-7f-1, let-7f-2, let-7g, miR-30a), this trend does not match the clinical trend. The clinical outcome presented by the KM survival plot shows that these miRNAs are associated with a low hazard ratio (HR < 1.0), which is consistent with a protective biomarker (colored yellow). Furthermore, for 8 SF-miRNAs whose expression level decreased with increased cellular carcinogenesis (miR-6087, miR-1246, miR-7704, miR-3604, miR-622, miR-612, miR-5047, miR-7161), the clinical results are consistent with poor survival rate (colored purple). Note that the calculated HR and the logrank statistics for miR-622 and miR-7704 are exceptionally high (HR is 2.23). For the other five SF-miRNAs, no significant difference is associated with clinical survival rates (e.g., miR-221). Importantly, the majority of the strongly differentially expressed SF-miRNAs (13) show inconsistency between the expression trend in SF-miRNA and the clinical outcomes, suggesting that these miRNAs, in the nucleus, act on different targets to those in the cytoplasm. Only miR-21 and miR-222 are consistent with the role of these miRNAs as oncomiRs, and the miR-148a as a tumor suppressor.

It should be noted that the SF-miRNAs discussed in Figure 8 and Figure 9 are not restricted to mature miRNAs. We tested whether the occurrences of segments other than the mature miRNAs from the SF (Supplemental Table S4) may explain the observed discrepancy in the expression trend with respect to miRNAs in the cytoplasm. We observed that 7 out of the 9 SF-miRNAs, which consist of mature miRNAs (>96%) in MCF-10A and MDA-MB-231, show such discrepancy (miR-100, miR-30a, let-7g, let-7i, let-7f-2 in both external comparisons (Figure 8 and Figure 9) and miR-7704 and miR-1246 (Figure 9). For all these 7 SF-miRNAs we argue that it is not the miRNA processing responsible for the discrepancy between the total and spliceosomal miRNA changes in levels, but most likely it reflects pairing with different targets in the spliceosome.

## 3. Discussion

miRNAs are mainly known for their role in translation inhibition in the cytoplasm [3,4,5,6,7]. The finding of miRNAs in the nucleus [17,18,19,20,21] led to the discovery of novel functions for miRNAs in this cell compartment [22,23]. Nuclear miRNAs are involved in the regulation of non-coding RNAs (ncRNAs), in transcriptional silencing, activation, and in inhibition (reviewed in refs. [20,21]). However, the functions of miRNAs in the nucleus are not yet well understood, and require further studies to elucidate their full potential [21]. One plausible hypothesis for the origin of miRNAs in the nucleus concerns the localization of miRNA genes in introns. In such instances, the biogenesis is likely linked to the endogenous spliceosome and accompanies the expression of the miRNA host genes. However, we have shown that SF-miRNAs are not enriched in intronic positioning [54,55]. While previous studies of nuclear miRNAs analyzed the whole nuclear miRNA population, our study focuses on miRNAs enriched in the SF. The identification of a large collection of SF-miRNAs in HeLa cells [54] together with the accumulating reports on deregulation of miRNAs in cancer [1], and particularly in breast cancer [2,68], have directed us to investigate the composition and expression profiles of SF-miRNAs in established cell-lines that are often used as in an in-vitro cellular breast-cancer model.

Female breast cancer is the second most commonly diagnosed cancer and the fifth leading cause of cancer-related death worldwide [2,69,70,71,72]. Oncogenic and tumor suppressor-like functions for miRNAs affect cell proliferation, apoptotic response, and metastasis in breast cancer [2]. However, the potential of SF-miRNAs to contribute to the properties of the breast cancer cellular models has not been studied before, and is of utmost importance. In this study we compare the SF-miRNAs of a healthy breast origin cell-line (MCF-10A) with the estrogen-dependent MCF-7 cell-line and the metastatic estrogen-independent MDA-MB-231 cells. While any cell-line cannot fully capture the complexity of the tissue, the MDA-MB-231 cells share features of the triple-negative breast cancer (TNBC) and display epithelial to mesenchymal transition (EMT) associated with metastasis. Several studies applied overexpression or suppression of specific cytosolic miRNAs to alter the cellular properties and affect major cellular signaling network [73], which in turn altered the capacity of these cells for invasion, migration, and apoptosis [74,75,76].

The presence of miRNAs in the SF suggests their potential in having nuclear functions. Bioinformatic analysis for targets of SF-miRNAs through base-pairing, highlighted the prospect of such sequences to regulate gene expression and splicing [54]. Here we focused on miR-7704, which is complementary to sequences of the first exon of the lncRNA HAGLR (Figure 5). We showed that low expression of SF-miR-7704 is detected in the highly malignant cell (Table 1, Figure 5) and it is negatively correlated with the expression of HAGLR. We further demonstrated that manipulating cells by suppressing the expression level of miR-7704 negatively regulates HAGLR expression (Figure 6). The direct impact of miR-7704 on HAGLR expression corroborate the findings made in HeLa cells [54]. Notably, HAGLR is an lncRNA that was implicated in a number of cancer types and was shown to be upregulated in different human cancers including bladder, cervical, colorectal, gastric, ovarian, prostate cancers, glioma, hepatocellular carcinoma, melanoma, osteosarcoma, and non-small cell lung cancer [56]. HAGLR has been shown to play a role in the development and progression of these cancers, and its expression level was correlated with cancers’ clinical features. Although the presence of HAGLR was not previously reported in breast cancer [56], we demonstrate here that its highest expression level among the three tested cell lines was found in MDA-MB-231. These cells are used as a model for aggressive, TNBC-like metastatic breast cancer. We have thus identified HAGLR as the nuclear target of miR-7704, which acts as a tumor suppressor in the spliceosomal context. In testing the impact of miR-7704 expression on the pathology and cancer survival, it was recently shown that a lower expression of miR-7704 is associated with more advanced stages of gastric cancer and a higher risk of liver metastasis in rectal cancer [77]. The showcase of the miR-7704 in SF, its impact on gene expression of the lncRNA HAGLR, and other cellular properties argues for the potential of SF-miRNAs from different cells to become a valuable resource for biomarker and a new target discovery.

Sequencing of the SF small RNA from three breast-derived cell lines revealed a rich collection of miRNA-derived sequences that signified each of the cells and indicated a unique profile. Among the SF-miRNAs we identified known cancer-associated oncomiRs (e.g., miR-21, miR-221, miR-222) and several tumor suppressor-like miRNAs (e.g., miR-100, miR-612, miR-30a and several members of the let-7 family; Table 1). Importantly, we found that the expression of many of these cancer-related miRNAs signify each of the discussed cell lines (Figure 7). These changes are portrayed not only in the level of expression (Table 1, Figure 2, Figure 3 and Figure 7), but also in the composition of the segmental regions of expressed SF-miRNAs (Figure 4). We observed that the SF-miRNA sequences are not only confined to mature miRNAs. Actually, only ~25% of SF-miRNAs are made up of mature miRNAs in the non-malignant MCF-10A cells, but this fraction reaches 60% and 68% in MCF-7 and MDA-MB-231, respectively (Figure 4B). These observations probably underlie the processing of the miRNA-related sequences as reflected by the difference in length distribution in each cell-line (Figure 4C). Yet, these changes in partition of the miRNA segmental regions between the cell-lines, and especially between the breast cancer cell-lines (MCF-7 and MDA-MB-231) and the non-malignant cell-line MCF-10A, likely reflect the potential of SF-miRNAs to differentially modify their targets.

Lastly, we monitored the large changes in the level of expression of SF-miRNAs across the 3 cell-lines (Figure 2, Figure 3 and Figure 7). The unexpected discrepancy between the tendency of miRNA expression in healthy versus cancerous breast cancer data from literature (i.e., miRCancer) compared to SF-miRNAs in the breast cell-lines is intriguing. In these cases, it is most likely that the SF-miRNAs might engage in base-pairing with nuclear targets that are different from those in the cytosol (Figure 8 and Figure 9). On the one hand, we detect miRNAs in which the expression trend is consistent with the literature (e.g., miR-21, miR-221, miR-222, miR-148a, and miR-622). However, for the set of other major abundant miRNAs (let-7g, let-7i, let-7f-2, let-7f-1, miR-30a, and miR-100), the SF-miRNAs show an opposite trend to that of the literature, and were signified by an increase in expression in breast cancer. Moreover, the expression of miR-1246, which is known as a tumorigenic-associated miRNA, in the spliceosome is suppressed in cells that resemble a malignancy state. The impact of miRNA expression in breast cancer biopsies and the survival rate from cohorts with thousands of samples allowed us to get closer to the clinical relevance of our findings. Recall that the characteristics in each database that we analyzed by the KM plots are clinically different. While the follow-up for women with breast cancer in TCGA is on average 25 months, it is much longer for METABRIC (94 months). Despite these differences, we found that the results confirmed and substantiated the discrepancy between the expression trend of some SF-miRNAs and the observation reported from the literature (based on miRCancer), showing a high degree of consistency versus SF-miRNAs among the literature summary and clinical samples. Notably, the majority of the SF-miRNAs show an inverse expression trend in view of the total miRNA analysis (13 of 21 informative miRNAs). The others are either below statistical significance, and only 3 SF-miRNAs are consistent with the clinical findings (Figure 9). We thus postulate that the majority of significant and highly expressed SF-miRNAs act on alternative targets in the spliceosome as compared with their targets in the cytoplasm, leading to an inverse impact on malignancy. Specifically, several known oncogenic miRNAs act as tumor suppressors in the spliceosome, while several tumor suppressors miRNAs increase tumorigenic characteristics in the spliceosome. Thus, further study might reveal overlooked nuclear targets that can be attractive biomarkers. We conclude that the expression of the abundant SF-miRNAs (22 miRNAs) could serve as indicators for the cancerous state of cells and breast cancer samples.

## 4. Materials and Methods

### 4.1. Plasmids

For overexpression of hsa-mir-7704, the pre-miR-7704 sequence (5′-CGGGGTCGGCGGCGACGTGCTCAGCTTGGCACCCAAGTTCTGCCGCTCCGACGCCCGGC-3′) was cloned into the pPRIME-CMV-GFP-FF3 vector at the Xho1 and EcoR1 sites by GENEWIZ (South Plainfield, NJ, USA), generating pPRIME-hsa-mir-7704-CMV-GFP-FF3 plasmid [54]. A pPRIME-CMV-GFP-FF3 empty vector was used as a control.

### 4.2. Cells

Spliceosomes and RNA were isolated from the following cell-lines: MCF-10A, non-tumorigenic human mammary epithelial cell-line (ATCC, Manassas, VA, USA, CRL-10317); MCF-7 mammary epithelial adenocarcinoma cell-line (ATCC HTB-22); MDA-MB-231 aggressive metastatic tumorigenic human mammary epithelial cell-line (ATCC HTB-26).

### 4.3. Isolation of Supraspliceosomes

All isolation steps were conducted at 4 °C. Supraspliceosomes were prepared from nuclear supernatants enriched in supraspliceosomes as previously described [51] from the following cell-lines: MCF-10A, MCF-7 and MDA-MB-231. Briefly, nuclear supernatants were prepared from purified cell nuclei by microsonication of the nuclei and precipitation of the chromatin in the presence of an excess of tRNAs. The nuclear supernatant was fractionated on 10%–45% (*v*/*v*) glycerol gradients. Centrifugations were carried out at 4 °C in an SW41 rotor run at 41 krpm for 90 min (or an equivalent ω^2^t = 2500 (ω is in krpm; t is in h)). The gradients were calibrated using the tobacco mosaic virus as a 200S sedimentation marker. Supraspliceosome peak fractions were confirmed by Western blot (WB) and by electron microscopy visualization.

### 4.4. Protein Detection

WB analyses were performed as previously described [59]. We used anti-hnRNP G (kindly provided by Prof. Stefan Stamm, University of Kentucky, Lexington), visualized with horseradish peroxidase conjugated to affinity-pure Goat anti-Rabbit IgG (H+L; Jackson Immunoreaserch, West Grove, PA, USA, 1:5000).

### 4.5. RNA Isolation from Supraspliceosomes and Deep Sequencing

RNA was extracted as previously described [51] from supraspliceosomes prepared from each of the different three breast cell-lines: the two breast cancer cells at different stages of malignancy: MCF-7 and MDA-MB-231; and the non-tumorigenic breast cell-line MCF-10A. The integrity of the RNA was evaluated by an Agilent 2100 bioAnalyzer. For small RNA library construction, ~1 µg of RNA was used. After phosphatase and T4 polynucleotide kinase (PNK) treatments, the RNA was ethanol-precipitated to enrich for small RNA, and small RNA libraries (in triplicates) were prepared according to NEBNext Small RNA Library Prep Set for Illumina (Multiplex Compatible) Library Preparation Manual. Adaptors were then ligated to the 5′ and 3′ ends of the RNA, and cDNA was prepared from the ligated RNA and amplified to prepare the sequencing library. The amplified sequences were purified on E-Gel^®^ EX 4% Agarose gels (ThermoFisher, Waltham, MA, USA, # G401004), and sequences representing RNA smaller than 200 nt were extracted from the gel. The library was sequenced using the Illumina NextSeq 500 Analyzer. The sequencing data, after removal of the adaptors and after filtering out low quality sequences, were aligned to mirBase (Release 21). In addition, the filtered high-quality fragments were mapped to the human transcriptome of hg19 gtf file from UCSC provided by Galaxy. The hg19 transcriptome contains 963,559 exons from 45,314 transcripts. Sequences aligned to the miRNA genes as compiled in miRBase are reported.

### 4.6. Next Generation Sequencing (NGS) Analysis

RNA was extracted from three independent biological preparations from the supraspliceosome fractions from each of the different breast cell-lines. NGS was performed for each sample on small RNA (<200 nt) molecules using standard Illumina Protocol. Each library consisted of an average of 18.5M (±9 M) reads of maximum length 76 (see Supplementary Table S1).

Raw data of the sequenced small RNA were trimmed using Cutadapt ver. 1.13. Low-quality reads were filtered out using FASTX toolkit. Reads from the SF were aligned against human genome hg19 and the miRbase database (version 21) using TopHat 2.1.1, allowing 90% sequence identity and a maximum of two mismatches. Reads whose start and end positions were mapped to miRNA genes were considered. High quality reads from the three SF preparations of each cell-line were combined. Out of the mapped reads, only reads of length ≥ 17 were considered. miRNA gene-aligned sequences refer to all mapped, high quality reads that are aligned to any of the pre-miRNA as defined by miRBase. For the rest of the analyses, only miRNA aligned-sequences with ≥10 reads per cell-line were considered. This threshold was used to ensure a reliable support in view of the limited total reads assigned from supraspliceosomes. The analyzed data were added to the ENA and the data accession number is PRJEB40524.

For cell lines, comparisons of the mapped reads were converted to RPM (reads per million) and biases due to data inflation were addressed using the DEseq2 package [78]. The DEseq2 builds a model for the observed counts, using a normalization parameter, for differences in library size, a variance parameter, and parameters representing the group differences. The fit for those parameters is done by using maximum likelihood estimations. The different values are also set according to the amount of information available for each gene. According to the false discovery rate (FDR), the changes in the gene differences are set and a significance LRT (likelihood ratio test) was applied. Only miRNAs that reached significant differential expression statistical values were further analyzed.

### 4.7. Validation of Gene Expression

#### 4.7.1. RT-PCR

RT-PCR was performed on RNA extracted from the cell-lines described above, and from the nuclear supernatants of the above cells as described [60]. The following sets of primers for HAGLR was used: Forward (exon 1) 5′-CGTCGGAGCGGCAGAACTT-3′ and Reverse 5′-AAGGGCCCATTTTCAGGCCA-3′ (exon 2). The primers for HOXD1 are: Forward (exon 1) 5′-ATTTACCTCCGGCTCACTCG-3′ and Reverse: 5′-AGGTGCAAGCAGTTGGCTAT-3′ (exon 2). The identity of all PCR products was confirmed by sequencing. Each experiment was repeated at least 3 times. The relative abundance was quantified in view of the intensity of the ß-actin that was used as a control. The ß-actin Forward and Reverse primers, for an amplicon of 140 nt, are: 5’-CTGGAACGGTGAAGGTGACA-3’ and 5’-AAGGGACTTCCTGTAACAATGCA-3’, respectively.

#### 4.7.2. Transfection and RNA Isolation

MCF-10A, MCF-7 and MDA-MB-231 cells were each grown in six-well plates. For downregulation of hsa-mir-7704, the cells were transfected with Anti-hsa-mir-7704 inhibitor AM29132 (ThermoFisher, Waltham, MA, USA) according to the manufacturer’s instructions at 100 nM for 48 h. As controls we used the same cells transfected with mirVana miRNA inhibitor and non-treated cells. The mirVana miRNA inhibitor (negative control #1; AM17010, Ambion, Austin, TX, USA) is a random sequence Anti-miR molecule that has been extensively tested in human cell lines and proved to have no identifiable effects on miRNA function.

Nuclear RNA isolation was performed as previously described [44]. Briefly, 48 h post transfection, the six-well plates were washed with PBS followed by the addition of 175 μL of cold RLN buffer (50 mM Tris pH 8, 140 mM NaCl, 1.5 mM MgCl_2_ and 0.5% NP40). The cells were then scraped and moved to an Eppendorf tube on ice for 5 min. Centrifugation for 2 min at 300× *g*, at 4 °C, was then performed. The supernatant was transferred to a new tube and the pellet (nuclei) was centrifuged again. RNA was then extracted from nuclei with miRNeasy mini kit (Qiagen, Hilden, Germany), following the manufacturer’s instructions. All experiments were performed with at least three biological replicates.

For overexpression of miR-7704, the MDA-MB-231 cells were grown in six-well plates and transfected for 48 h with pPRIME-miR-7704-CMV-GFP-FF3 plasmid (5.5 μg/well) using Lipofectamine-2000 reagent (ThermoFisher). As control, we used transfection with 5.5 μg/well of the empty vector. Removal of adherent cells from the plates was according to routine protocol (using Trypsin, and PBS with 10% FCS to inactivate it) 36 h post transfection. Cell suspension was transferred to the FACS tube and stained with 5 μL Hoechst (1 mg/mL, Sigma #33342) as a measure for DNA cell content. Cell cycle analysis was performed using a fluorescence analysis (using FACS BD-Facsaria III Bactlab) equipped with 405 nm and 488 nm lasers, for Hoechst-A and GFP, respectively. Gating applied for all live cells in the preparation. FACS data analysis used Kaluza and the Beckman Coulter Flow Cytometry Analysis Software.

### 4.8. Quantitative PCR

#### 4.8.1. TaqMan microRNA Assay

For RT-PCR of miR-7704, the TaqMan Advanced miRNA cDNA synthesis kit (ThermoFisher) was used according to the manufacturer’s instructions, which included polyA tailing, adaptor ligation and miR-Amp reaction as previously described [54].

#### 4.8.2. RT of mRNA

RT of nuclear RNA was performed using the High Capacity cDNA Reverse Transcription Kit (ThermoFisher) according to the manufacturer’s instructions, using RT Random Primers, and MultiScribeTM Reverse Transcriptase.

#### 4.8.3. Quantitative PCR Reaction

mRNA and miR-7704 levels were measured using the TaqMan Fast Advanced Mix (ThermoFisher) and the following TaqMan Assays with FAM/MGB-NFQ primers/probe: TaqMan Advance MiR Assay hsa-mir-7704, 480576_mir (AB-25576, ThermoFisher); TaqMan Gene Expression Assays MTO, XS/PC: beta actin: Hs99999903_m1 (AB-4453320); TaqMan Gene Expression Assays /PC: HOXD1: Hs04334671_g1 (AB-4448892); Custom TaqMan Copy Number Assays, SM/PC: HAGLR_NR_033979.2 (AB-4400294). Fw primer: TGCCAAGCTGAGCACGTC, Rev: TACTCCAGATCTGGGGAC, FAM Probe: ACGTACTCCAGATCTG. Assays were performed according the manufacturer’s instructions. Amplification was carried out using a QuantStudio 12K Flex Real-Time PCR System (for downregulation), and StepOnePlus Real-Time PCR System (for overexpression), for 40 cycles at annealing temperature of 60 °C. Analysis was performed using the delta-delta C_T_, 2^−ΔΔ*C*t^ method. All experiments were performed in at least three biological repeats.

## 5. Conclusions

Comparison of the miRNA sequences associated with the endogenous spliceosome of human breast-derived cell-lines that range in their cancerous states (from the non-malignant MCF-10A, the malignant MCF-7, and the highly metastatic MDA-MB-231 cells), revealed changes with malignancy of the rich collection of SF-miRNAs, including changes in the miRNA types, level of expression, and the composition of pre-miRNA segmental regions.

Notably, most abundant and differentially expressed SF-miRNAs in the three breast cell lines (e.g., miR-100, miR-30a, and let-7 family members) exert an expression trend that is different from that reported in the literature and in breast cancer clinical samples. One such miRNA is miR-7704 whose genomic position overlaps HAGLR, a cancer-related lncRNA. In the three tested breast cell-lines, we quantified an inverse expression of miR-7704 with respect to HAGLR, with miR-7704 expression associated with a reduced malignancy. Moreover, inhibiting miR-7704 expression led to an increased level of HAGLR, while its overexpression led to an alteration in cell cycle in MDA-MB-231 cells. While miR-7704 is reported as oncomiR in breast cancer patients, it acts as a tumor suppressor in the SF context, with HAGLR being its nuclear target. The negative regulation of HAGLR by miR-7704 can serve as an overlooked path in controlling aggressive breast cancer. The findings of numerous miRNAs that apparently act differently in the cytosol and in the SF context implies that these SF-miRNAs act on yet unexplored targets in the nucleus. Altogether, we highlight miRNAs in the spliceosome as an unexplored route in resolving the molecular mechanisms of breast cancer.

## Figures and Tables

**Table 1:** Highly expressed 25 SF-miRNAs.

miRNA ^a^	Mean MCF10	Mean MCF7	Mean MDA	Trend ^b^ MCF7/MCF10	Trend ^b^ MDA/MCF10	Genomic Annotation	Cluster ^c^
hsa-mir-6087 *	5847.1	1124.3	705.3	D	D	Intergenic	
hsa-mir-21	362.6	1173.3	1155.5	U	U	Exon, 3′UTR	
hsa-mir-1246	484.6	83.8	32.0	D	D	Intron, LncRNA	
hsa-mir-3687 *	460.6	34.9	8.2	D	D	Ribosomal RNA	
hsa-mir-100	13.1	0.0	483.4	D	U	Intron, LncRNA	hsa-mir-526; hsa-let-7a-2
hsa-mir-612	450.5	1.6	3.0	D	D	LncRNA	
hsa-mir-7704	296.4	41.8	20.2	D	D	Exon, LncRNA	
hsa-let-7f-1	54.0	90.6	184.7	U	U	Intergenic	**hsa-let-7a-1**; hsa-let-7d
hsa-let-7f-2	32.1	86.0	173.2	U	U	Intergenic	**has-mir-98**
hsa-let-7g	20.4	84.8	170.6	U	U	Intron, Coding	
hsa-mir-3064	196.6	46.3	8.8	D	D	Mirtron, Coding	**hsa-mir-5047**
hsa-mir-1291 **	88.0	147.6	14.9	U	D	Intron, Coding	
hsa-let-7i	21.2	62.3	140.3	U	U	Mirtron, LincRNA	
hsa-mir-5047	163.8	14.5	1.2	D	D	Intron, Coding	**hsa-mir-3064**
hsa-mir-622	54.6	74.2	16.8	U	D	Intergenic	
hsa-mir-19b-1	14.0	60.1	50.6	U	U	LncRNA	hsa-mir-17; hsa-mir-18a; hsa-mir-19a; **hsa-mir-20a**; **hsa-mir-92a-1**
hsa-mir-20a	15.1	53.2	51.0	U	U	LncRNA	hsa-mir-17; hsa-mir-18a; hsa-mir-19a; **hsa-mir-19b-1**; **hsa-mir-92a-1**
hsa-mir-222	6.5	0.0	105.5	D	U	Intron, LncRNA	**hsa-mir-221**
hsa-mir-30a	5.2	0.8	91.1	D	U	Intron, LincRNA	
hsa-mir-148a	47.4	32.4	4.0	D	D	Intergenic	
hsa-mir-7161	74.7	1.9	1.2	D	D	Intron, Coding	
hsa-mir-24-1	27.1	10.2	40.3	D	U	Intron, Coding	**hsa-mir-23b**; **hsa-mir-27b**; hsa-mir-3074
hsa-mir-1248 **	66.8	4.5	5.3	D	D	Intron, Coding	
hsa-mir-3607 **	57.0	10.8	4.3	D	D	Intron, Coding	
hsa-mir-221	8.5	0.0	59.7	D	U	Intron, LncRNA	**hsa-mir-222**

^a^ miRNAs marked by * were removed from the recent version of miRbase (ver22.0); miRNAs marked by ** overlap with SNORA/D. ^b^ Expression trend: U, D depicts up and down, respectively. The trend represents the relative level of miRNA expression relative to MCF-10A. ^c^ Cluster neighbors are miRNAs within a genomic distance < 10 k, as defined by miRBase. Marked in bold are cluster neighbor miRNAs, which are found among SF-miRNAs (supported by >10 reads). MCF-10A (MCF10), MCF-7 (MCF7), MDA-MB-231 (MDA).

## Supplementary Materials

Supplementary Materials can be found at https://www.mdpi.com/1422-0067/21/21/8132/s1. Figure S1. Spearman rank correlation between all 9 samples of the SF-miRNA collection identified in each sample. Table S1. A statistical summary of the sequencing of breast cell-lines libraries. Table S2. Amounts of aligned reads from SF-miRNAs (raw data) associated with each of the nine sample preparations from three cell-lines. Source data for Figure 2 and Figure S1. Table S3. A list of SF-miRNAs according to their statistical significance following normalization by DESeq2. Source for Figure 3, Table 1 and Figure 7. Table S4. List of SF-miRNA according to segmental regions of a prototype of pre-miRNAs for each of the tested cell-lines. A summary for the segmental region partition for cell-lines. Source data for Figure 4. Table S5. Comparison of changes of 22 SF-miRNAs in the breast cell-lines with data from literature (miRCancer). Source data for Figure 8. Table S6. Comparison of changes of 22 SF-miRNAs in the breast cell-lines with breast cancer KM survival plots. Source data for Figure 9.

## Abbreviations

EMT	Epithelial to mesenchymal transition
FDR	False discover rate
HP-miRNA	hairpin precursor miRNA
KM	Kaplan-Meier
lncRNA	Long non-coding RNA
SF	Spliceosomal fraction
TNBC	Triple-negative breast cancer
WB	Western blot
