# Supplementary material for: Spliceosome-Associated microRNAs Signify Breast Cancer Cells and Portray Potential Novel Nuclear Targets
Source: Int J Mol Sci. 2020 Oct 30;21(21):8132. doi: 10.3390/ijms21218132 (PMC7663234; doi:10.3390/ijms21218132)
Supplement: Supplementary file 1 [file ijms-21-08132-s001.pdf]

SUPPLEMENTARY MATERIALS

Spliceosome-Associated microRNAs Signify Breast Cancer Cells and Portray Potential Novel Nuclear targets

Shelly Mahlab-Aviv [1], Keren Zohar [1], Yael Cohen[2], Ayelet R. Peretz[2], Tsiona Eliyahu[1], Michal Linial[1,*] and Ruth Sperling[2,*]

Figure S1. Spearman rank correlation between all 9 samples of the SF-miRNA collection identified in each sample.

Table S1. A statistical summary of the sequencing of breast cell-lines libraries.

Table S2. Amounts of aligned reads from SF-miRNAs (raw data) associated with each of the nine sample preparations from three cell-lines. Source data for **Figure** 2 and **Supplemental Figure S1**.

Table S3. A list of SF-miRNAs according to their statistical significance following normalization by DESeq2. Source for **Figure 3**, **Table** 1 and **Figure 5**.

Table S4. List of SF-miRNA according to segmental regions of a prototype of pre-miRNAs for each of the tested cell-lines. A summary for the segmental region partition for cell-lines. Source data for **Figure 4**.

Table S5. Comparison of changes of 22 SF-miRNAs in the breast cell-lines with data from literature (miRCancer). Source data for **Figure 8**.

Table S6. Comparison of changes of 22 SF-miRNAs in the three breast cell-lines with breast cancer KM survival plots. Source data for **Figure 9**.

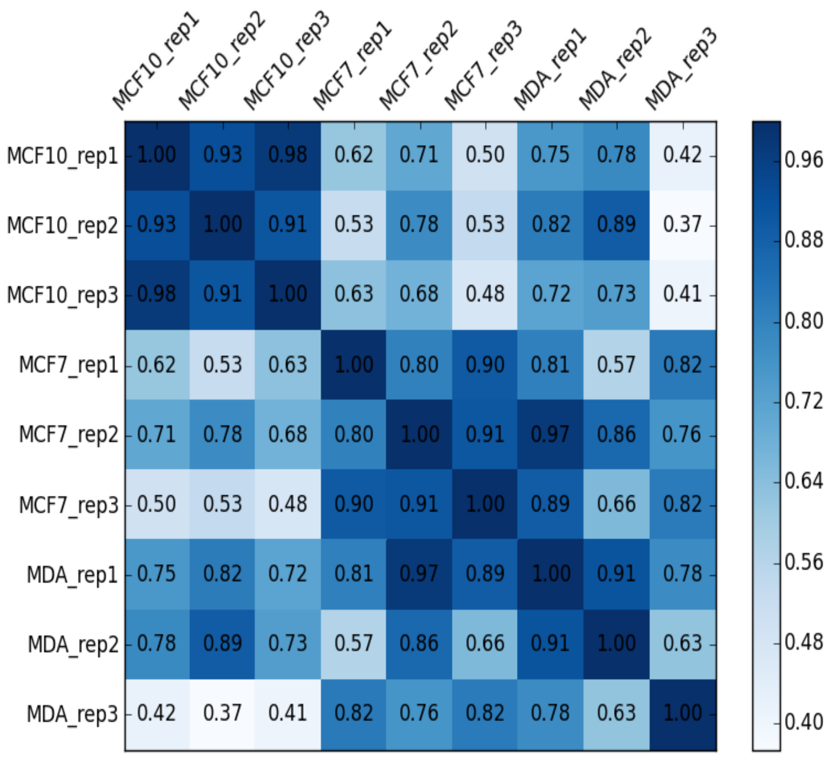

Figure S1. Spearman rank correlation between all 9 samples of the SF-miRNA collection identified in each sample.

Table S1: Sequencing data for the breast cancer cell lines and replicates

Cell Replicates	Total reads	Mapped reads	% mapped reads	mapped to miRNA*	% of all mapped reads
MCF10A rep1	31150316	19173084	61.6	10611	0.055343
MCF10A rep2	33718453	20087790	59.6	17053	0.084892
MCF10A rep3	25493755	14013958	55	8611	0.061446
MCF7 rep1	10716584	2242497	20.9	3687	0.164415
MCF7 rep2	6492651	1725734	26.6	1882	0.109055
MCF7 rep3	17231509	2600045	15.1	2957	0.113729
MDA-231 rep1	11529138	2852117	24.7	4078	0.142982
MDA-231 rep2	17987596	10005933	55.6	10571	0.105647
MDA-231 rep3	11800122	2141920	18.2	6293	0.293802

* Reads of length >= 17 and at most 2 alignment to the genome

Table S2: The sum of reads associated with each of the three cell-line. Threshold >=10 reads

Reads>10	SUM_MCF10_TH_10_READS	SUM_MCF7_TH_10_READS	SUM_MDA_TH_10_READS
hsa-mir-21	1293	2496	5644
hsa-mir-6087	20334	2422	3343
hsa-mir-100	45	0	2487
hsa-let-7f-1	190	198	927
hsa-let-7f-2	112	187	860
hsa-let-7g	73	191	695
hsa-let-7i	76	138	661
hsa-mir-222	23	0	527
hsa-mir-30a	19	0	473
hsa-mir-221	29	0	349
hsa-mir-20a	47	110	239
hsa-mir-19b-1	43	127	238
hsa-mir-92a-1	33	36	188
hsa-mir-24-2	44	16	181
hsa-mir-24-1	95	18	178
hsa-mir-92a-2	28	15	157
hsa-mir-1246	1814	189	144
hsa-mir-27b	53	0	136
hsa-mir-26a-2	42	22	119
hsa-mir-26a-1	45	22	118
hsa-mir-30d	45	65	100
hsa-mir-7704	1167	80	94
hsa-mir-1307	16	43	84
hsa-mir-622	195	162	78
hsa-mir-103a-1	36	17	76
hsa-mir-151a	37	31	75
hsa-mir-103a-2	39	20	74
hsa-mir-25	10	35	73
hsa-let-7b	61	21	71
hsa-mir-99b	23	37	69
hsa-mir-4426	126	14	66
hsa-mir-423	27	38	65
hsa-mir-125b-1	0	0	64
hsa-mir-1291	312	340	59
hsa-mir-320a	12	25	56
hsa-mir-23a	14	0	53
hsa-mir-10a	0	0	52
hsa-mir-125b-2	0	0	51
hsa-mir-3687	1595	71	46
hsa-mir-3064	642	105	42
hsa-mir-98	16	13	40
hsa-mir-29a	27	0	39
hsa-mir-30c-1	12	0	39
hsa-mir-30c-2	0	0	39
hsa-mir-182	38	71	36
hsa-mir-15a	42	27	33
hsa-mir-3654	91	22	33

hsa-mir-148b	0	0	33
hsa-mir-191	15	0	32
hsa-mir-22	15	0	32
hsa-mir-425	0	0	30
hsa-mir-181a-1	45	0	29
hsa-mir-6515	152	27	28
hsa-mir-584	0	0	27
hsa-mir-146a	0	0	26
hsa-mir-92b	0	0	26
hsa-mir-224	22	0	25
hsa-mir-183	15	31	24
hsa-mir-3607	185	26	24
hsa-mir-27a	11	0	24
hsa-let-7d	0	0	24
hsa-let-7e	66	24	23
hsa-mir-1248	255	10	23
hsa-mir-4709	57	0	21
hsa-mir-30e	0	0	21
hsa-mir-200b	0	27	20
hsa-mir-3198-2	35	13	20
hsa-mir-612	1494	0	20
hsa-mir-128-1	0	0	20
hsa-mir-23b	57	0	19
hsa-mir-17	0	0	18
hsa-mir-492	0	49	17
hsa-mir-574	0	0	17
hsa-mir-93	16	13	16
hsa-mir-125a	25	0	16
hsa-mir-148a	170	69	15
hsa-mir-378c	13	11	15
hsa-mir-18a	0	0	15
hsa-mir-744	0	15	14
hsa-mir-7705	27	0	14
hsa-mir-6819	16	0	14
hsa-mir-181a-2	0	0	14
hsa-mir-16-2	0	0	14
hsa-mir-6840	85	21	13
hsa-mir-106b	0	10	13
hsa-mir-29b-2	41	0	13
hsa-mir-198	11	0	13
hsa-mir-99a	0	0	13
hsa-mir-16-1	0	0	13
hsa-mir-140	0	0	12
hsa-mir-7-1	0	0	12
hsa-let-7a-2	0	0	12
hsa-mir-3609	0	20	11
hsa-mir-26b	10	0	11
hsa-mir-7110	0	0	11
hsa-mir-126	0	0	11
hsa-mir-361	0	0	11

hsa-mir-671	43	0	10
hsa-mir-4761	30	0	10
hsa-mir-181b-2	0	0	10
hsa-mir-101-1	0	0	10
hsa-mir-218-1	0	0	10
hsa-mir-5047	542	33	0
hsa-mir-200c	71	32	0
hsa-mir-200a	0	20	0
hsa-mir-4664	63	16	0
hsa-mir-6811	0	15	0
hsa-mir-296	0	11	0
hsa-mir-7161	249	0	0
hsa-mir-205	153	0	0
hsa-mir-147b	138	0	0
hsa-mir-3648	132	0	0
hsa-mir-6849	111	0	0
hsa-mir-6887	77	0	0
hsa-mir-555	77	0	0
hsa-mir-1306	70	0	0
hsa-let-7a-1	63	0	0
hsa-mir-663a	62	0	0
hsa-mir-4461	52	0	0
hsa-mir-3916	51	0	0
hsa-mir-8075	49	0	0
hsa-mir-614	46	0	0
hsa-mir-664a	41	0	0
hsa-mir-7706	37	0	0
hsa-mir-6504	36	0	0
hsa-let-7a-3	36	0	0
hsa-mir-6775	36	0	0
hsa-mir-640	35	0	0
hsa-mir-3605	29	0	0
hsa-mir-663b	28	0	0
hsa-mir-4485	27	0	0
hsa-mir-4723	25	0	0
hsa-mir-6866	24	0	0
hsa-mir-4680	24	0	0
hsa-mir-1204	22	0	0
hsa-mir-4712	21	0	0
hsa-mir-626	21	0	0
hsa-mir-922	21	0	0
hsa-mir-6764	20	0	0
hsa-mir-5689	20	0	0
hsa-mir-3652	19	0	0
hsa-mir-6837	19	0	0
hsa-mir-6865	19	0	0
hsa-mir-4647	19	0	0
hsa-mir-6742	18	0	0
hsa-mir-29c	18	0	0
hsa-mir-937	18	0	0

hsa-mir-6848	18	0	0
hsa-mir-4730	17	0	0
hsa-mir-6851	16	0	0
hsa-mir-6510	16	0	0
hsa-mir-4687	16	0	0
hsa-mir-7113	16	0	0
hsa-mir-645	15	0	0
hsa-mir-378h	15	0	0
hsa-mir-6824	15	0	0
hsa-mir-197	15	0	0
hsa-mir-8086	15	0	0
hsa-mir-4435-1	14	0	0
hsa-mir-4435-2	14	0	0
hsa-mir-647	14	0	0
hsa-mir-4700	14	0	0
hsa-mir-3118-2	14	0	0
hsa-mir-4701	14	0	0
hsa-mir-6723	13	0	0
hsa-mir-3940	13	0	0
hsa-mir-877	12	0	0
hsa-mir-5088	12	0	0
hsa-mir-3198-1	12	0	0
hsa-mir-571	12	0	0
hsa-mir-6501	12	0	0
hsa-mir-623	12	0	0
hsa-mir-1825	12	0	0
hsa-mir-4477b	12	0	0
hsa-mir-4673	11	0	0
hsa-mir-3651	11	0	0
hsa-mir-2110	11	0	0
hsa-mir-452	10	0	0
hsa-mir-545	10	0	0
hsa-mir-6516	10	0	0
hsa-mir-632	10	0	0
hsa-mir-590	10	0	0
hsa-mir-2355	10	0	0
hsa-mir-378d-2	10	0	0
hsa-mir-4640	10	0	0
hsa-mir-8066	10	0	0
hsa-mir-644a	10	0	0
hsa-mir-6859-2	10	0	0
hsa-mir-3614	10	0	0
hsa-mir-4800	10	0	0
hsa-mir-6859-1	10	0	0

Table S3. Statistically significant DESeq2 analysis

miRNA	baseMean	log2FoldChange	lfcSE	stat	pvalue	padj
hsa-mir-6087	2558.89802	-3.0521954	0.49515467	43.6657604	3.30E-10	**4.74E-09**
hsa-mir-21	897.111909	1.67098614	0.3170605	31.8807457	1.19E-07	**1.06E-06**
hsa-mir-1246	200.157887	-3.9417058	0.5636884	48.9613135	2.33E-11	**3.97E-10**
hsa-mir-100	165.493465	5.22001233	0.41573157	306.722586	2.49E-67	**4.65E-65**
hsa-mir-3687	167.8572	-5.7728652	0.72686862	60.6722535	6.69E-14	**1.39E-12**
hsa-mir-612	151.688222	-7.026533	0.66299282	161.196522	9.92E-36	**9.28E-34**
hsa-mir-7704	119.490449	-3.8877571	0.7597268	28.3284397	7.06E-07	**5.07E-06**
hsa-let-7f-1	109.765431	1.77147391	0.32407778	30.7989887	2.05E-07	**1.67E-06**
hsa-mir-3064	83.8800941	-4.497302	0.5844071	53.6927804	2.19E-12	**4.10E-11**
hsa-let-7f-2	97.11638	2.43060472	0.34569813	47.6459745	4.51E-11	**7.02E-10**
hsa-let-7g	91.9128119	3.05378696	0.57657209	25.0038973	3.72E-06	**2.58E-05**
hsa-mir-5047	59.8564654	-6.7981308	0.77072614	96.1243485	1.34E-21	**6.26E-20**
hsa-let-7i	74.5873251	2.71177463	0.46710245	32.0203392	1.11E-07	**1.04E-06**
hsa-mir-222	37.3028832	4.01289566	0.53763086	126.009282	4.34E-28	**2.70E-26**
hsa-mir-1291	83.5139685	-2.6325279	0.52837151	35.8479575	1.64E-08	**1.71E-07**
hsa-mir-30a	32.3578899	4.10061265	0.67499463	73.6398571	1.02E-16	**2.73E-15**
hsa-mir-7161	25.9661352	-5.700116	0.81883928	75.1278723	4.86E-17	**1.51E-15**
hsa-mir-1248	25.5229935	-3.7093356	0.73681834	37.0434482	9.04E-09	**1.06E-07**
hsa-mir-622	48.5234195	-1.7411792	0.45331477	22.4968147	1.30E-05	**8.70E-05**
hsa-mir-221	22.7372138	2.83353003	0.60822213	66.8132576	3.10E-15	**7.25E-14**
hsa-mir-24-1	25.8488677	0.5606365	0.65763848	8.07251605	0.01766345	**0.04587589**
hsa-mir-20a	39.7818953	1.78265597	0.66753775	8.2620934	0.01606605	**0.04231482**
hsa-mir-19b-1	41.5747357	1.88088387	0.73600475	8.29326586	0.01581759	**0.04225555**
hsa-mir-3607	24.0448261	-3.6474937	0.66821249	32.9904856	6.86E-08	**6.75E-07**
hsa-mir-6515	22.3309069	-2.7896507	0.80619426	12.9807118	0.00151801	**0.00550476**
hsa-mir-24-2	20.5646833	1.73067526	0.65982852	12.9274075	0.00155901	**0.00550476**
hsa-mir-148a	27.933263	-3.7922436	0.62182069	35.8773707	1.62E-08	**1.71E-07**
hsa-mir-4426	18.4857731	-1.3369762	0.52533812	17.0521214	0.00019823	**0.00092675**
hsa-mir-92a-1	21.1078471	1.96318601	0.58790355	12.1170093	0.00233789	**0.00794884**
hsa-mir-147b	13.4614296	-5.684554	0.95144271	76.1180195	2.96E-17	**1.11E-15**
hsa-mir-92a-2	15.6407616	1.90848473	0.57718836	17.5499736	0.00015455	**0.00080281**
hsa-mir-27b	14.0284887	0.62402176	0.70110245	10.6109094	0.00496444	**0.01600604**
hsa-mir-6849	12.7276706	-4.5778464	0.96352422	30.9490671	1.90E-07	**1.62E-06**
hsa-mir-3654	13.565232	-2.3331341	0.77781859	9.81539163	0.0073895	**0.02228767**
hsa-mir-6840	13.3780225	-3.221585	0.79398987	16.1463208	3.12E-04	**0.00138824**
hsa-mir-1306	8.55367674	-4.3451392	1.08536659	20.7513788	3.12E-05	**0.0001993**
hsa-mir-555	8.71815176	-5.7080609	1.16346843	38.1809995	5.12E-09	**6.38E-08**
hsa-mir-1307	14.5196638	1.99800785	0.73747112	9.55295047	0.00842565	**0.02423993**
hsa-mir-4709	7.35738428	-1.8379158	0.83783876	13.0933749	0.00143486	**0.00536638**
hsa-let-7a-1	7.5568804	-3.5114896	0.89654823	20.115463	4.29E-05	**0.0002585**
hsa-mir-23b	7.3719498	-2.4809388	0.99669054	11.0930113	0.00390107	**0.01279823**
hsa-mir-200c	11.5571139	-5.6434554	1.22012687	30.6993039	2.16E-07	**1.68E-06**
hsa-mir-663a	6.26593046	-5.3421453	1.2547554	29.9559166	3.13E-07	**2.34E-06**
hsa-mir-182	16.072265	-0.6584059	0.61301425	15.5761506	0.00041465	**0.00180325**
hsa-mir-3916	6.83768061	-3.0392145	0.8962524	14.0660583	0.00088226	**0.00351025**
hsa-mir-25	10.6307751	2.11393896	0.7641579	10.1488088	0.00625481	**0.01949416**
hsa-mir-671	5.89671512	-2.3466503	0.98156171	9.59291573	0.00825895	**0.02413162**
hsa-mir-29a	5.37515051	-0.0325122	0.86181301	14.2828141	0.00079164	**0.00321818**
hsa-mir-4461	6.7136104	-4.3286761	1.12156324	17.0539698	1.98E-04	**0.00092675**
hsa-mir-8075	5.23661261	-5.3795268	1.51443751	19.9690959	4.61E-05	**0.00026944**
hsa-mir-29b-2	4.74801898	-2.5682561	1.05484747	17.1310994	0.00019056	**0.00092675**
hsa-mir-614	4.97604822	-3.6571646	1.0190484	20.7012305	3.20E-05	**0.0001993**
hsa-mir-125b-1	4.1894403	2.42495624	1.03116321	17.3768209	0.00016853	**0.00085175**
hsa-mir-224	3.96486423	-0.4680097	1.03875754	10.0238372	0.00665812	**0.02041095**

hsa-mir-6775	4.19329523	-5.5523401	1.66277778	13.3180793	0.00128238	**0.00489397**
hsa-mir-664a	4.1638028	-3.8771537	1.20838349	15.4982575	0.00043112	**0.00180739**
hsa-mir-10a	4.13335409	3.53845327	1.16786508	13.8395466	0.00098805	**0.00384929**
hsa-mir-4723	3.0341914	-3.1993155	1.37623385	11.8397824	0.00268549	**0.00896763**
hsa-mir-3605	2.966161	-5.2481077	1.61903352	17.6583134	0.0001464	**0.0007822**
hsa-mir-663b	2.90468425	-4.1177551	1.43105377	15.4806364	0.00043493	**0.00180739**
hsa-mir-183	7.98651124	0.19847746	0.80884006	7.8869112	0.01938113	**0.04964754**
hsa-mir-125b-2	2.7294829	3.83019682	1.5848312	12.9259161	0.00156017	**0.00550476**
hsa-mir-4485	2.59642701	-4.9728624	1.58895595	16.2769656	0.00029208	**0.00133217**
hsa-mir-4680	2.25153631	-4.2003124	1.61331845	12.5794238	0.00185529	**0.00642482**
hsa-mir-922	2.21129961	-3.6903587	1.67397767	9.61761314	0.00815759	**0.02413162**
hsa-mir-6848	2.07279728	-3.84454	1.70554097	9.46328768	0.00881197	**0.02496726**
hsa-mir-3652	2.91661523	-4.5999267	1.6331325	8.40654442	0.01494659	**0.04050742**
hsa-mir-29c	1.89668112	-3.9444462	1.74527479	9.39845678	0.0091023	**0.02540492**
hsa-mir-584	1.89561165	3.08258121	1.61301866	8.60089766	0.01356247	**0.03729679**
hsa-let-7d	2.66152338	4.85209462	1.55898666	10.3174	0.00574917	**0.01822194**
hsa-mir-492	9.29818331	3.43663808	1.36591068	41.6775322	8.91E-10	**1.19E-08**
hsa-mir-200b	5.73063218	3.48205995	1.4565434	19.1914229	6.80E-05	**0.00037411**
hsa-mir-200a	3.58050777	0.91656333	1.70335893	19.2977725	6.45E-05	**0.00036549**

Table S4. Fraction of Mature of the other aligned reads by segmental region

	MCF10A	MCF7	MDA-231
extension	13.89187	9.078925	8.727222
mature	25.44188	60.04101	68.23237
undefined complement	0.985612	1.026544	0.626379
hairpin precursor	3.027587	2.240941	1.245125
overlap region	56.65305	27.61258	21.1689

Removed extension that are beyond the complete hairpin pre-miRNA

Table S5. SF-miRNA expression in view of miRcancer

Names	padj	Aver MCF-10A	Aver MCF-7	Aver MDA-MB-231	ratio MDA/MCF-10A	ratio MCF-10A/MDA
hsa-mir-612	9.28E-34	450.45	1.57	3.05	0.007	147.920
hsa-mir-5047	6.26E-20	163.84	14.51	1.21	0.007	135.210
hsa-mir-7161	1.51E-15	74.73	1.94	1.23	0.016	60.874
hsa-mir-3687	1.39E-12	460.56	34.85	8.16	0.018	56.466
hsa-mir-3064	4.10E-11	196.59	46.28	8.78	0.045	22.395
hsa-mir-1246	3.97E-10	484.63	83.84	32.00	0.066	15.144
hsa-mir-7704	5.07E-06	296.40	41.85	20.23	0.068	14.654
hsa-mir-3607	6.75E-07	56.98	10.82	4.34	0.076	13.138
hsa-mir-1248	1.06E-07	66.78	4.54	5.25	0.079	12.719
hsa-mir-148a	1.71E-07	47.42	32.41	3.97	0.084	11.930
hsa-mir-6087	4.74E-09	5847.12	1124.30	705.28	0.121	8.291
hsa-mir-1291	1.71E-07	88.03	147.59	14.91	0.169	5.903
hsa-mir-622	8.70E-05	54.58	74.17	16.81	0.308	3.246
hsa-mir-21	1.06E-06	362.56	1173.27	1155.51	3.187	0.314
hsa-let-7f-1	1.67E-06	54.02	90.58	184.70	3.419	0.292
hsa-let-7f-2	7.02E-10	32.11	85.99	173.25	5.395	0.185
hsa-let-7i	1.04E-06	21.19	62.31	140.25	6.618	0.151
hsa-mir-221	7.25E-14	8.52	0.00	59.69	7.007	0.143
hsa-let-7g	2.58E-05	20.37	84.80	170.57	8.372	0.119
hsa-mir-222	2.70E-26	6.46	0.00	105.45	16.328	0.061
hsa-mir-30a	2.73E-15	5.23	0.77	91.07	17.397	0.057
hsa-mir-100	4.65E-65	13.06	0.00	483.42	37.014	0.027

Names	total MCF-10A+MDA	miRCancer evidence	Down (Cancer to healthy)	Up (Cancer to healthy)	our finding SF-miRNA	% Consistancy within miRCancer
hsa-mir-612	453.50	2	2	0	down	1.000
hsa-mir-5047	165.06	0	0	0	down	NA
hsa-mir-7161	75.96	0	0	0	down	NA
hsa-mir-3687	468.72	0	0	0	down	NA
hsa-mir-3064	205.36	1	1	0	down	1.000
hsa-mir-1246	516.63	10	3	7	down	0.3
hsa-mir-7704	316.63	0	0	0	down	NA
hsa-mir-3607	61.32	2	1	1	down	0.500
hsa-mir-1248	72.03	0	0	0	down	NA
hsa-mir-148a	51.39	52	46	6	down	0.885
hsa-mir-6087	6552.40	0	0	0	down	NA
hsa-mir-1291	102.95	3	2	1	down	0.667
hsa-mir-622	71.40	9	7	2	down	0.778
hsa-mir-21	1518.07	268	7	261	up	0.026
hsa-let-7f-1	238.72	14	14	0	up	1
hsa-let-7f-2	205.36	14	14	0	up	1.000
hsa-let-7i	161.45	17	17	0	up	1.000
hsa-mir-221	68.21	98	9	89	up	0.092
hsa-let-7g	190.94	20	19	1	up	0.950
hsa-mir-222	111.91	61	6	55	up	0.098
hsa-mir-30a	96.30	51	45	6	up	0.882
hsa-mir-100	496.48	39	34	5	up	0.872

At least 75% of evidence must be consistent. Otherwise marked as mixed evidence.

Table S6. Expression trend for SF-miRNAs in breast cell lines and KM survival plots

SF-miRNA (22 genes)	miRNA MDA > MCF10[a]	HR	(95% confidence interval)	Logrank P-value	Data from KM Survival Plots[b]	Expected change from KM[c]	Inverse trend SF & KM plot
hsa-mir-6087	D	2	1.43-2.79	3.8e-05	OncomiR	U	Opposite
hsa-mir-21	U	1.63	1.16-2.27	0.004	OncomiR	U	Consistent
hsa-mir-1246	D	1.52	1.23-1.87	9e-05	OncomiR	U	Opposite
hsa-mir-3687	D	-	-	-	Not listed	-	
hsa-mir-100	U	0.7	0.57-0.85	0.00032	Tumor sup.	D	Opposite
hsa-mir-612	D	2.06	1.48-2.87	1.5e-05	OncomiR	U	Opposite
hsa-mir-7704	D	2.23	1.59-3.12	2.1e-06	OncomiR	U	Opposite
hsa-let-7f-1	U	0.72	0.59-0.87	0.00079	Tumor sup.	D	Opposite
hsa-let-7f-2	U	0.72	0.59-0.87	0.00079	Tumor sup.	D	Opposite
hsa-let-7g	U	0.72	0.59-0.88	0.0014	Tumor sup.	D	Opposite
hsa-mir-3064	D	1.69	1.2-2.39	0.0025	OncomiR	U	Opposite
hsa-mir-1291	D	1.35	0.96-1.9	0.088	Not significant	n.s.	
hsa-let-7i	U	0.82	0.66-1.01	0.065	Not significant	n.s.	
hsa-mir-5047	D	2.23	1.59-3.12	2.1e-06	OncomiR	U	Opposite
hsa-mir-622	D	2.23	1.59-3.13	2e-06	OncomiR	U	Opposite
hsa-mir-3607	D	0.71	0.49-1.04	0.08	Not significant	n.s.	
hsa-mir-1248	D	1.39	0.97-1.99	0.069	Not significant	n.s.	
hsa-mir-222	U	1.35	1.07-1.71	0.013	OncomiR	U	Consistent
hsa-mir-30a	U	0.75	0.61-0.91	0.0041	Tumor sup.	D	Opposite
hsa-mir-148a	D	0.7	0.57-0.86	0.00062	Tumor sup.	D	Consistent
hsa-mir-7161	D	2.2	1.57-3.06	3.3e-06	OncomiR	U	Opposite
hsa-mir-221	U	1.24	0.87-1.76	0.23	Not significant	n.s.	

[a]Expression of SF-miRNA in MDA-MB-231 relative to MCF-10A cells. Trend is annotated U or D for up and down, respectively. [b]Data from KM survival plot for miRNAs from these cell lines. [c]Expected trend in these cell-lines as extracted from KM survival plots, where oncogenic miRNAs are annotated as U (up) while tumor suppressor miRNAs are expected to go down (D). miRNAs that do not exhibit a significant trend are marked as non-significant (n.s.). SF-miRNAs that result in inverse annotations for the cellular view and patients' survival are marked as opposite.
